# Treatment Challenges of a Primary Vertebral Artery Aneurysm Causing Recurrent Ischemic Strokes

**DOI:** 10.1155/2017/2571630

**Published:** 2017-01-10

**Authors:** Davide Strambo, Luca Peruzzotti-Jametti, Aurora Semerano, Giovanna Fanelli, Franco Simionato, Roberto Chiesa, Enrico Rinaldi, Vittorio Martinelli, Giancarlo Comi, Marco Bacigaluppi, Maria Sessa

**Affiliations:** ^1^Stroke Unit, Department of Neurology and Neurophysiology, San Raffaele Scientific Institute, Via Olgettina 60, 20132 Milan, Italy; ^2^Neuroimmunology Unit, Institute of Experimental Neurology, San Raffaele Scientific Institute, Via Olgettina 60, 20132 Milan, Italy; ^3^Neuroradiology Department, San Raffaele Scientific Institute, Via Olgettina 60, 20132 Milan, Italy; ^4^Vascular Surgery Department, San Raffaele Scientific Institute, Via Olgettina 60, 20132 Milan, Italy

## Abstract

*Background*. Extracranial vertebral artery aneurysms are a rare cause of embolic stroke; surgical and endovascular therapy options are debated and long-term complication may occur.* Case Report*. A 53-year-old man affected by neurofibromatosis type 1 (NF1) came to our attention for recurrent vertebrobasilar embolic strokes, caused by a primary giant, partially thrombosed, fusiform aneurysm of the left extracranial vertebral artery. The aneurysm was treated by endovascular approach through deposition of Guglielmi Detachable Coils in the proximal segment of the left vertebral artery. Six years later the patient presented stroke recurrence. Cerebral angiography and Color Doppler Ultrasound well characterized the unique hemodynamic condition developed over the years responsible for the new embolic event: the aneurysm had been revascularized from its distal portion by reverse blood flow coming from the patent vertebrobasilar axis. A biphasic Doppler signal in the left vertebral artery revealed a peculiar behavior of the blood flow, alternately directed to the aneurysm and backwards to the basilar artery. Surgical ligation of the distal left vertebral artery and excision of the aneurysm were thus performed.* Conclusion*. This is the first described case of NF1-associated extracranial vertebral artery aneurysm presenting with recurrent embolic stroke. Complete exclusion of the aneurysm from the blood circulation is advisable to achieve full resolution of the embolic source.

## 1. Introduction

Vertebral aneurysms are an unusual cause of posterior circulation strokes, far less frequent than other embolic sources. While posterior circulation aneurysms are mostly located in the intracranial circulation, extracranial aneurysms are exceedingly rare. The best treatment option is controversial [1, 2] since both surgical and endovascular procedures carry technical difficulties. Altering the hemodynamics of vertebrobasilar circulation may lead to long-term complications that are difficult to forecast. Here we report an exemplary case of a primary, giant, fusiform aneurysm of the left vertebral artery (VA) causing recurrent embolic strokes of the posterior circulation. After a first endovascular procedure to exclude the aneurysm the patient developed a unique hemodynamic condition responsible for further embolization that required surgical intervention.

## 2. Clinical Presentation

**Figure 1:**
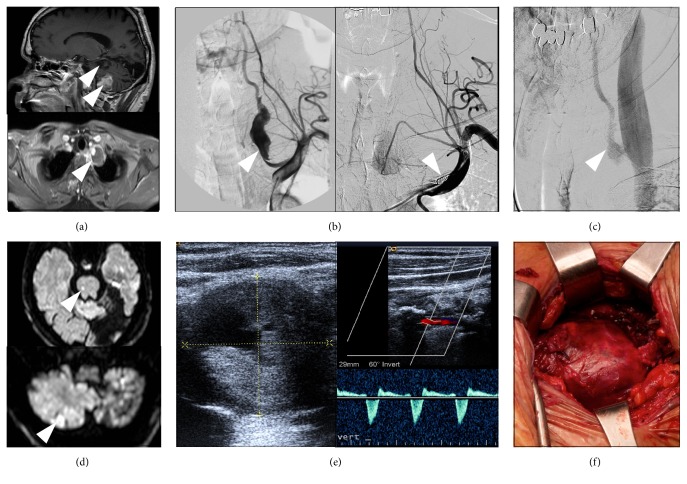
(a) (Top) sagittal T1-weighted contrast enhanced brain magnetic resonance imaging (MRI) scan showing a subacute ischemic lesion of the inferior left cerebellar hemisphere in the left posterior-inferior cerebellar artery (PICA) territory and chronic cerebellar and occipital ischemic lesions; (bottom) axial T1-weighted contrast enhanced MRI image of the neck showing a giant fusiform aneurysm (arrow), containing an eccentric thrombotic formation, originating from the left vertebral artery. (b) Left subclavian angiograms showing (left) the displasic and tortuous aspect of the giant aneurysm and (right) aneurysm exclusion after the endovascular treatment by deposition of GDC vortex spirals in the proximal segment of left vertebral artery (VA) (arrow). (c) Late sequences of right vertebral angiogram performed six years after the endovascular procedure showing full revascularization of the rostral part of the aneurysm by retrograde blood flow from the patent vertebrobasilar axis. (d) Diffusion-weighted imaging (DWI) brain MRI showing new acute ischemic lesions of the cerebellum and the brainstem (arrows). (e) Sonography and Color Doppler Ultrasound of neck vessels showing the aneurysm (3.5 cm) and the biphasic flow in the left vertebral artery supportive of the embolic etiology of the new ischemic stroke. (f) Surgery of the giant aneurism by aneurysmorraphy with thrombectomy: aneurysm exposure and isolation from the surrounding tissue.

A 53-year-old white man affected by a sporadic form of neurofibromatosis 1 (NF1) [3] came to our attention five days after the acute onset of nausea, vomiting, and unsteadiness of gait. The patient had suffered from a left occipital ischemic stroke four years earlier. Our neurological evaluation revealed dysarthria, right side paresthesias, and left limbs ataxia in addition to right lateral homonymous hemianopia that was secondary to the previous ischemic event. Brain Magnetic Resonance Imaging (MRI) showed subacute ischemic lesion in the left cerebellar hemisphere and bilateral cerebellar and occipital chronic ischemic lesions (Figure 1(a)). Major cardioembolic sources, alterations of the coagulation cascade, and mitochondrial diseases were excluded. Intracranial MRI angiography was normal, while neck-MRI and angiography of cervical vessels disclosed a primary fusiform 4 cm wide aneurysm originating from the V1 portion of the left VA and containing an eccentric intraluminal thrombotic formation (Figure 1(a)), highly suggestive of being responsible of the multiple ischemic strokes. On cerebral angiography, the parent artery just proximal to the aneurysm presented a severe focal stenosis (diameter of 1.5 mm) and had a normal diameter of 5 mm (comparable to the right VA) in its segment distal to the aneurysm.

To resolve the embolic source and prevent new ischemic strokes, a surgical intervention was considered of second choice due to the high risk of cerebral embolization and high mortality rates reported after VA ligation [4]. Therefore, an endovascular procedure was performed with deposition of three Guglielmi Detachable Coils (GDC) 18-vortX® (2 mm × 4 cm, 2 mm × 5 cm, and 2 mm × 3 cm) and three GDC-10 360 (3 mm × 6 cm soft), in the proximal segment of left VA, aimed to interrupt the orthograde blood flow from the aneurysm to the cerebral circulation (Figure 1(b)). The occlusion of the left VA distal to the aneurysm was not performed because it would have required either to direct the catheter through the partially thrombosed aneurysmal sac, with an unacceptable risk of distal embolism, or to navigate from the right VA to the vertebrobasilar junction and then downward through the left VA, which carried both technical difficulties and high risk of complications. In addition, having already interrupted the orthograde blood flow in the left VA, the benefits of these maneuvers in the prevention of new embolic strokes were uncertain.

The patient remained asymptomatic until six years later when he developed a new episode of acute gait unsteadiness, nausea, vomiting, and left side paresthesias, rapidly followed by transient loss of consciousness. Brain MRI revealed acute bilateral cerebellar and brainstem ischemic lesions (Figure 1(d)). Cerebral angiography showed revascularization of the aneurysm by reverse blood flow coming from the contralateral VA (Figure 1(c)). Cervical Color Doppler Ultrasound imaging disclosed a peculiar biphasic blood flow in the left VA directed alternately to the aneurysm and backwards to the basilar artery, which was the pathogenic mechanisms of the new embolic ischemic strokes (Figure 1(e)). Being the aneurysm excluded proximally by the previous endovascular procedure, surgical aneurysmorraphy with thrombectomy was now performed by ligation of distal left VA and complete excision of the aneurysm (Figure 1(f)). After two-year follow-up the patient is stable with no additional symptoms.

## 3. Discussion

The treatment of primary vertebral aneurysm is challenging due to its location, life-threating risk of interrupting blood supply to the basilar artery and rarity. Only nine patients have been described so far with extracranial VA aneurysms associated with NF1 and none of them presented with recurrent ischemic stroke due to embolization. However it is known that performing the sole proximal occlusion of the parent artery carries over time the risk of aneurysm revascularization by reverse blood flow coming from the patent vertebrobasilar axis [5], but, in previously described cases, this recanalization was asymptomatic except for one patient who developed a vertebral arteriovenous fistula [6].

The proximal occlusion of the left VA, initially performed in our patient, interrupted the orthograde blood flow carrying embolic material from the aneurysm to the cerebral circulation and was thus effective in the middle term to prevent new embolic strokes. However, the peculiar and unexpected biphasic flow established in the left VA still enabled the migration of embolic material originating from the aneurysm to the cerebral circulation. This eventuality has never been described before to our knowledge, but, in the treatment planning of extracranial VA aneurysms, it should be considered and possibly prevented to avoid the potentially inauspicious consequences of an ischemic stroke in the posterior circulation.

## 4. Conclusion

According to literature and to this paradigmatic case, in order to definitely exclude potential embolic sources and avoid long-term complications of aneurysm revascularization, both proximal and distal occlusions of the aneurysm sac have to be considered for extracranial VA aneurysms. Whenever complete definitive exclusion of the aneurysm cannot be achieved in a single endovascular intervention, a combined approach with surgical distal occlusion of the parent artery closely following the endovascular procedure is recommended.

## References

[1] Shang E. K., Fairman R. M., Foley P. J., Jackson B. M. (2013). Endovascular treatment of a symptomatic extracranial vertebral artery aneurysm. *Journal of Vascular Surgery*.

[2] Oderich G. S., Sullivan T. M., Bower T. C. (2007). Vascular abnormalities in patients with neurofibromatosis syndrome type I: clinical spectrum, management, and results. *Journal of Vascular Surgery*.

[3] Morasch M. D., Phade S. V., Naughton P., Garcia-Toca M., Escobar G., Berguer R. (2013). Primary extracranial vertebral artery aneurysms. *Annals of Vascular Surgery*.

[4] Shintani A., Zervas N. T. (1972). Consequence of ligation of the vertebral artery. *Journal of Neurosurgery*.

[5] Hiramatsu H., Matsui S., Yamashita S. (2012). Ruptured extracranial vertebral artery aneurysm associated with neurofibromatosis type 1. *Neurologia Medico-Chirurgica*.

[6] Ushikoshi S., Goto K., Uda K., Ogata N., Takeno Y. (1999). Vertebral arteriovenous fistula that developed in the same place as a previous ruptured aneurysm: a case report. *Surgical Neurology*.

